# Editorial: Agrobiodiversity at different scales for improving conservation strategies

**DOI:** 10.3389/fpls.2024.1457713

**Published:** 2025-01-08

**Authors:** Carmine Guarino, Guido Cipriani, Jesus V. Jorrin-Novo, Domenico Carputo, Claudio Di Vaio

**Affiliations:** ^1^ Department of Science and Technology, University of Sannio, Benevento, Italy; ^2^ Department of Agricultural, Food, Environmental and Animal Sciences, University of Udine, Udine, Italy; ^3^ Agroforestry and Plant Biochemistry, Proteomics and Systems Biology, Department of Biochemistry and Molecular Biology, University of Córdoba, Córdoba, Spain; ^4^ Department of Agricultural Sciences, University of Naples Federico II, Portici, Italy

**Keywords:** agrobiodiversity, plant conservation, resilience, agricultural landscape, genetic resources

This Research Topic is focused on different aspects of agrobiodiversity, whose multifaceted perspectives can give rise to many approaches in improving conservation strategies. This abundance and diversity of aims, scientific methodologies, and research approaches, characterize the works collected in this Research Topic. The strong interest in the topic is evident in some of the geographical areas coinciding with global biodiversity hotspots (Mediterranean and Central America). Here, studies have been conducted to enhance and conserve the biodiversity of great importance crops such as wheat (Taranto et al.), grapevine (Iorizzo et al.; Villano et al.), tomato (Donoso et al.) and potato (Dawson et al.). Other studies aimed to focus and preserve the natural historical heritage (Marchese et al.) and the cultural heritage (Tartaglia et al.). Classical approaches to the study of agrobiodiversity have been flanked by modern methodologies based on the analysis of big data useful in developing models for agrobiodiversity conservation on a large spatial and temporal time scales (Raggi et al.).

A first comprehensive analysis concerning the distribution across Italy of crop wild relatives, belonging to globally important genera, is presented by (Raggi et al.). The authors provide evidence on several crop wild relatives found *in situ* in a rather precarious condition. This is mainly due to the limited knowledge and/or limited number of populations of the most threatened species. Increasing efforts to identify taxa on field is crucial to develop effective protection actions dealing with the real needs of biodiversity conservation. While highlighting the great role of protected areas in conservation, the paper also points out the need of *ex-situ* conservation strategies. With a similar purpose, the work of Dawson et al. defines a methodology to identify priority sites for effective *on-farm* conservation of potato landraces. The consumption of wild edible plants, which is closely linked to the cultural history of a region, is part of people’s traditional and local identity, transcending mere food value. This issue is addressed by the work of Tartaglia et al., who analysed the traditionally consumed parts (fruit and leaves) of the strawberry tree (*Arbutus unedo* L.), an underutilised fruit tree typical of the Mediterranean region. From the metabolomic profile analysis of the matrices, the pedo-geographical imprinting, shaping the expression of metabolites on landscape characteristics, was evident. Furthermore, the metabolic variability observed in individuals of the same ecotype highlights the importance of environmental factors and agronomic practices in the geographical area of origin when addressing the issue of local agrobiodiversity.

The enhancement of historical agricultural landscapes and the protection of germplasm resources from genetic erosion are considered priorities by the international community. The Mediterranean basin is rich in monumental olive trees, found within important archaeological sites, and in rural areas. These long-lived trees can be a source of genes for resistance to biotic agents or even adverse climatic conditions that have occurred during their long lives. This gene pool is of great value and can be exploited by breeding programmes to produce new genotypes best suited to changing environmental conditions and emerging diseases such as *Xylella fastidiosa* subsp. *pauca* (ST53) Schaad et al.; it can also be used to achieve increasingly sustainable production. Since the value of old trees is intrinsic to the territory in which they are found, the protection of these plants is crucial. Marchese et al. place emphasis in their study on the selection of certain genotypes and genome resequencing projects to discover resilience traits against biotic and abiotic stresses.

Very interesting insights come from the work of Taranto et al., where an innovative correlation between genotypic and phenotypic traits of wheat accessions, useful for varietal registration, is shown. Results expand the knowledge about genetic architecture of many traits of agronomic interest, and pave the way for the use of genetic markers in current phenological descriptive protocols, thus improving the system of plant variety protection and registration. The article by Donoso et al. on the Chilean Limachino tomato (*Solanum lycopersicum* L.) also draws attention to the need for a careful characterisation of landraces by exploiting the genotypic and phenotypic peculiarities found in the Chilean tomato germplasm. Concerning the varietal characterisation through molecular techniques, of particular importance is the study of *Vitis vinifera* L. by (Villano et al.). Authors used whole-genome SNP datasets, generated by GBS and ddRADSeq methods, to assess the clonal diversity of six traditional grape varieties. The authors show how combining different SNP datasets is possible and useful for studying the inter- and intra-specific genetic diversity of grape populations. This is provided that the same reference genome is used. A repeatable framework was provided to optimise future computational studies based on retrieving information from partial analyses performed at different times and with different techniques. The results also demonstrated the value of advanced genomic methods in the study of population structure and the detection of synonymy/homonymy. The study by Iorizzo et al. focuses on the same species, but with significantly different aims. The authors investigate the impact of pedoclimatic conditions on the oenological performance of two grape varieties. They highlight how the phenotypic response of Aglianico and Cabernet Sauvignon vines is significantly influenced by the prevailing pedoclimatic conditions, in particular the physical properties of the soil.

In conclusion, this collection of papers highlights how diverse and multifaceted approaches should be encouraged and pursued to have an increasingly clear and comprehensive methodological framework leading to useful strategies for the conservation and enhancement of agrobiodiversity. Tackling the issue of agro-diversity on a global scale and with different scientific approaches is undoubtedly a viable avenue for further insights and perspectives.

